# Supplementary material for: Integrating unsupervised and reinforcement learning in human categorical perception: A computational model
Source: PLoS One. 2022 May 10;17(5):e0267838. doi: 10.1371/journal.pone.0267838 (PMC9089926; doi:10.1371/journal.pone.0267838)
Supplement: S1 File — We insert in this file many details regarding the model implementation (networks layers, hyper-parameters, task conditions, etc) and the computation of the results. (PDF) [file pone.0267838.s001.pdf]

Integrating unsupervised and reinforcement learning in human categorical perception: a computational model
SUPPLEMENTARY MATERIALS

Giovanni Granato [1,2] *, Emilio Cartoni [1], Federico Da Rold [3], Andrea Mattera [1], Gianluca Baldassarre [1],

[1] Laboratory of Computational Embodied Neuroscience, Institute of Cognitive Sciences and Technologies, National Research Council of Italy, Rome, Italy
[2] School of Computing, Electronics and Mathematics, University of Plymouth, Plymouth, U.K.
[3] Body Action Language Lab, Institute of Cognitive Sciences and Technologies, National Research Council of Italy, Rome, Italy,

* Corresponding author: giovanni.granato@istc.cnr.it

Abstract

Categorical perception identifies a tuning of human perceptual systems that can occur during the execution of a categorisation task. Despite the fact that experimental studies and computational models suggest that this tuning is influenced by task-independent effects (e.g., based on Hebbian and unsupervised learning, UL) and task-dependent effects (e.g., based on reward signals and reinforcement learning, RL), no model studies the UL/RL interaction during the emergence of categorical perception. Here we have investigated the effects of this interaction, proposing a system-level neuro-inspired computational architecture in which a perceptual component integrates UL and RL processes. The model has been tested with a categorisation task and the results show that a balanced mix of unsupervised and reinforcement learning leads to the emergence of a suitable categorical perception and the best performance in the task. Indeed, an excessive unsupervised learning contribution tends to not identify task-relevant features while an excessive reinforcement learning contribution tends to initially learn slowly and then to reach sub-optimal performance. These results are consistent with the experimental evidence regarding categorical activations of extrastriate cortices in healthy conditions. Finally, the results produced by the two extreme cases of our model can explain the existence of several factors that may lead to sensory alterations in autistic people.

1 Methods: further details of the model architecture, learning and simulations

Figure S1 shows a detailed computational schema of the architecture components.

Figure S2 shows a graphical representation of the restricted Boltzmann Machine training executed with the Contrastive Divergence algorithm [1].

We tested the model solving the sorting task with different task conditions (sorting rule, i.e. colour, shape or size) and perceptual component configurations (the number of

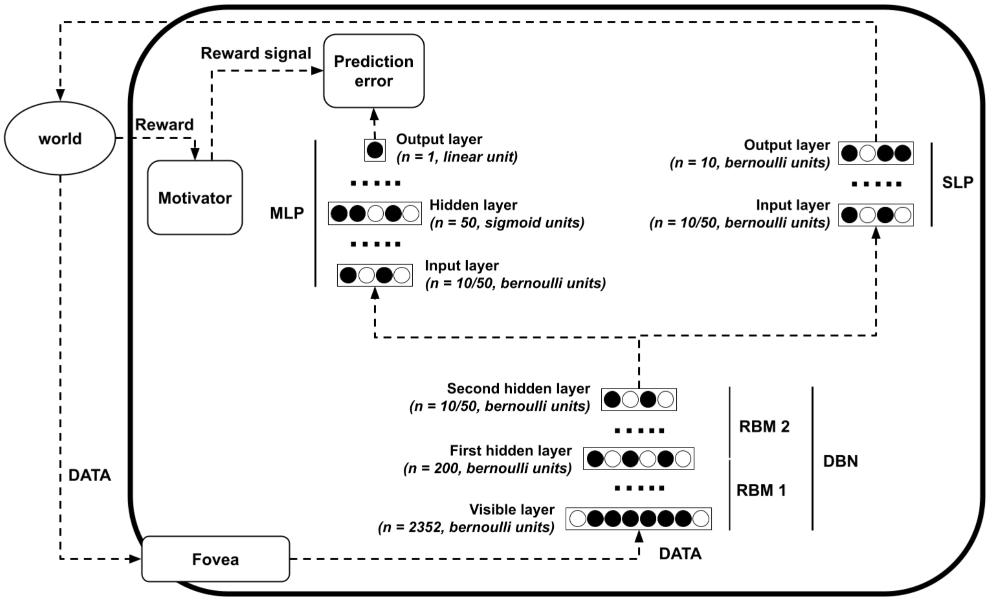

Figure S1. Detailed computational schema of the model. Each layer of the model is associated with the number and a type of the layer units. The dashed lines identify an information transmission without alterations (e.g. the activation of the second hidden layer of DBN corresponds to the activation of the input layer of the multi-layer perceptron and the single-layer perceptron. White boxes identify the non-neural components of the model. RBM: Restricted Boltzmann Machine; DBN: Deep Belief Network; SLP: Single-layer Perceptron; MLP: Multi-layers perceptron.

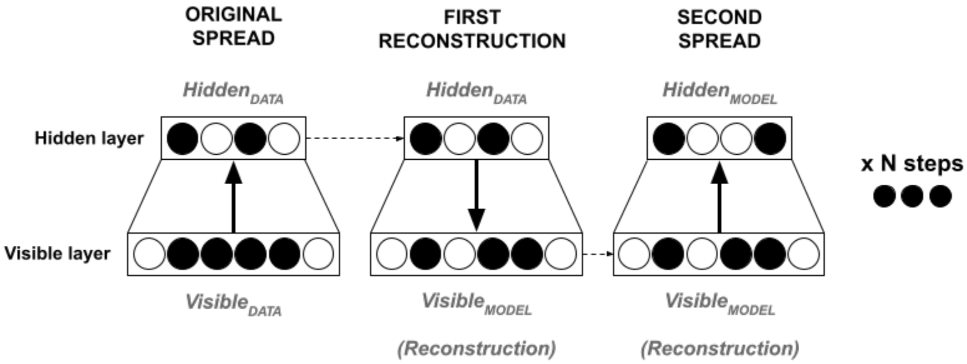

Figure S2. Graphical representation of the Restricted Boltzmann Machine training with the Contrastive Divergence. The dashed lines represent the start of a next step in the same network while the solid lines correspond to a network spread. Note that $Visible_{DATA}$ corresponds to the original input.

neurons of top hidden layer and the reward contribution into the learning process). We randomly changed these parameters, keeping the others fixed. Table S1 shows the key parameters of simulations.

Simulations parameters

Label	Value/Range	Description
Sorting rule	$\{colour, shape, size\}$	Variable.<br>latent rule to solve the sorting task
Training epochs	$15 * 10^3$	Fixed.<br>Training epochs of sorting task.
Single-layer perceptron output units	10	Fixed.<br>Output neurons of motor component.
Single-layer perceptron learning rate (REINFORCE)	$1 * 10^{-2}$	Fixed.<br>Training learning rate of motor component.
Multi-layers perceptron hidden units	50	Fixed.<br>Hidden neurons of predictor component.
Multi-layers perceptron learning rate (Backpropagation)	$1 * 10^{-3}$	Fixed.<br>Training learning rate of predictor component.
Single-layer perceptron learning rate (REINFORCE)	$1 * 10^{-2}$	Fixed.<br>Training learning rate of motor component.
DBN units (visible layer)	2352	Fixed.<br>Neurons of visible layer.
DBN units (first hidden layer)	200	Fixed.<br>Neurons of first hidden layer.
DBN units (second hidden layer)	$\{10, 50\}$	Variable.<br>Neurons of second hidden layer.
First RBM (off-line) training epochs	$1 * 10^3$	Fixed.<br>Training epochs necessary to achieve a dataset reconstruction error of 0.001.
First RBM learning rate (Constrastive Divergence)	$1 * 10^{-2}$	Fixed.<br>Training (offline) learning rate.
First RBM momentum (Constrastive Divergence)	0.9	Fixed.<br>Training (offline) momentum.
Second RBM learning rate (Constrastive Divergence)	$1 * 10^{-3}$	Fixed.<br>Training learning rate.
Second RBM momentum (Constrastive Divergence)	0.9	Fixed.<br>Training momentum.
Second RBM learning rate (REINFORCE)	$1 * 10^{-2}$	Fixed.<br>Training learning rate.
λ	$\{1, 0.1, 0.01, 0.001, 0\}$	Variable.<br>Contribution of the Contrastive Divergence to the weights update
Second RBM reward contribution	$(1 - \lambda)$,<br>with $\lambda \in \{1, 0.1, 0.01, 0.001, 0\}$	Variable.<br>Contribution of the REINFORCE to the weights update

Table S1. The table shows the simulations parameters.

2 Results: further statistical analysis

2.1 Performances analysis

Figures S3, S4, and S5 show the training curves of the models trained with different RL contributions in 15,000 epochs, for the three sorting categories and for the condition with 10 units of the DBN second hidden layer. Figures S6, S7, and S8 show the analogous curves for the condition involving 50 units of the DBN second hidden layer. In particular, these are the models of the condition with a high-level of computational resources, namely 50 units at the level of the second hidden layer of the DBN. In all conditions, the L0 models (with no reinforcement learning - RL, i.e. relying only on unsupervised learning - UL) show an initial highest performance with respect to the other models L1, L2, L3, and L4. This confirms that in L0 models representation learning is initially facilitated with respect to models with a higher RL contribution as the reward is initially erratic. Moreover, for all three category tasks the reward achieves a maximum final performance for the L2 models having a balanced level of UL and RL. Indeed, these models outperform the models with absent or very low RL (L0 and L1) because these employ a lot of computational resources for non-task specific features; moreover they outperform the models with very high or extreme RL (L3 and L4) because these tend to incur in local minima.

Learning curves of models: colour category, low computational resources

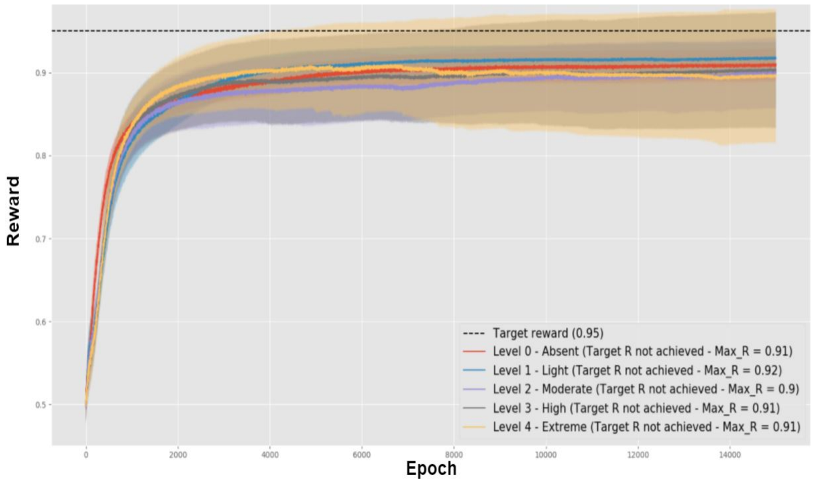

Figure S3. Reward per epoch in the task task involving the colour category and low computational resources, of the five models involving different UL/RL levels, averaged over the models using a given level. Shaded areas represent the curves standard deviations.

Learning curves of models: shape category, low computational resources

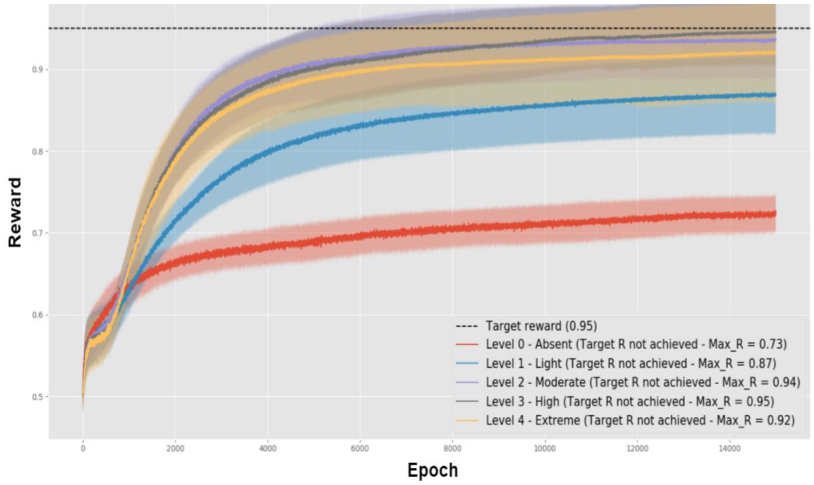

Figure S4. Reward per epoch in the task task involving the shape category and low computational resources, of the five models involving different UL/RL levels, averaged over the models using a given level. Shaded areas represent the curves standard deviations.

Learning curves of models: size category, low computational resources

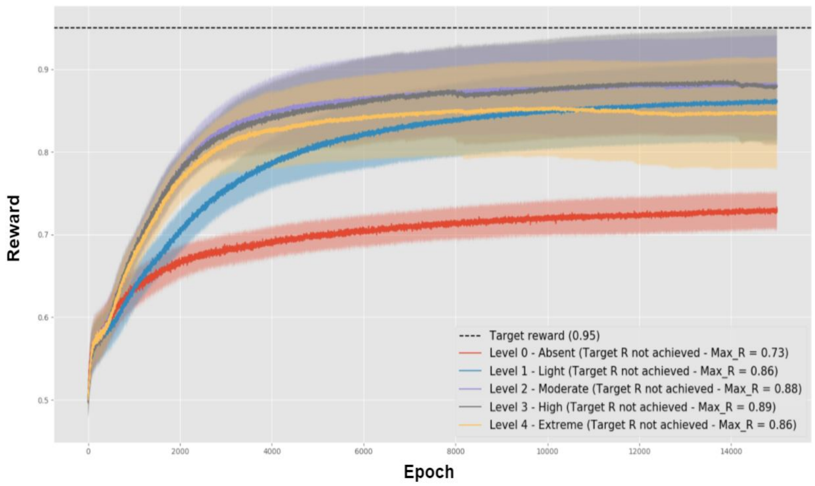

Figure S5. Reward per epoch in the task task involving the size category and low computational resources, of the five models involving different UL/RL levels, averaged over the models using a given level. Shaded areas represent the curves standard deviations.

Learning curves of models: colour category, high computational resources

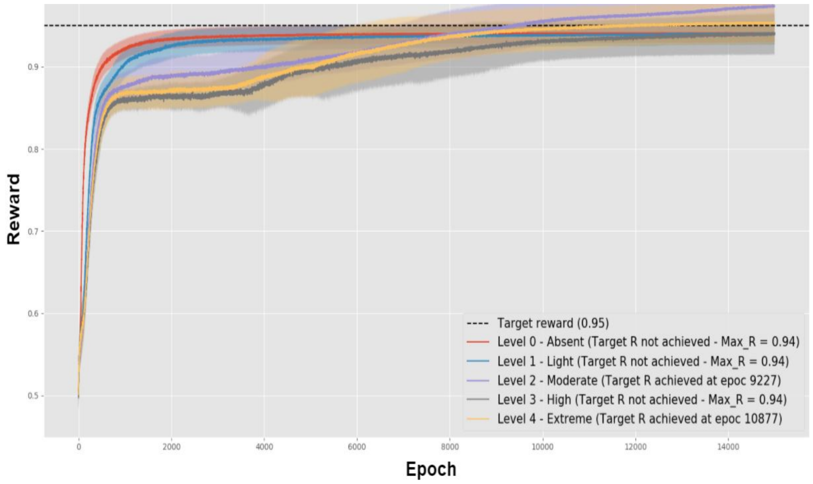

Figure S6. Reward per epoch in the task task involving the colour category and high computational resources, of the five models involving different UL/RL levels, averaged over the models using a given level. Shaded areas represent the curves standard deviations.

Learning curves of models: shape category, high computational resources

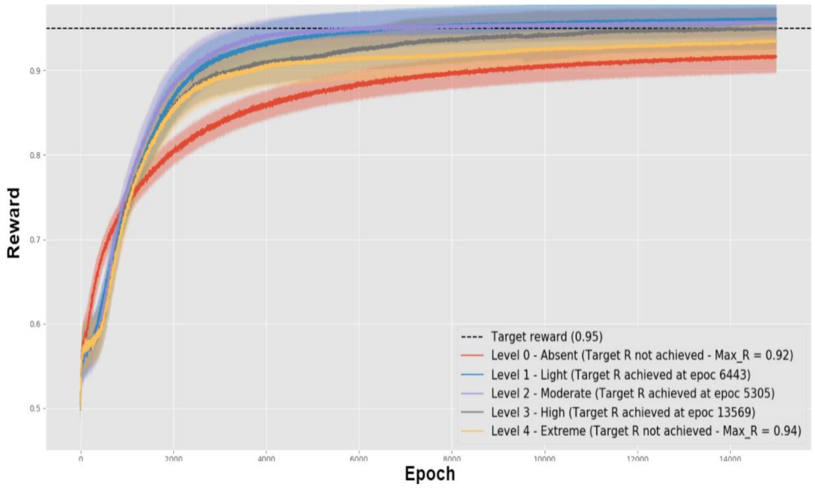

Figure S7. Reward per epoch in the task task involving the shape category and high computational resources, of the five models involving different UL/RL levels, averaged over the models using a given level. Shaded areas represent the curves standard deviations.

Learning curves of models: size category, high computational resources

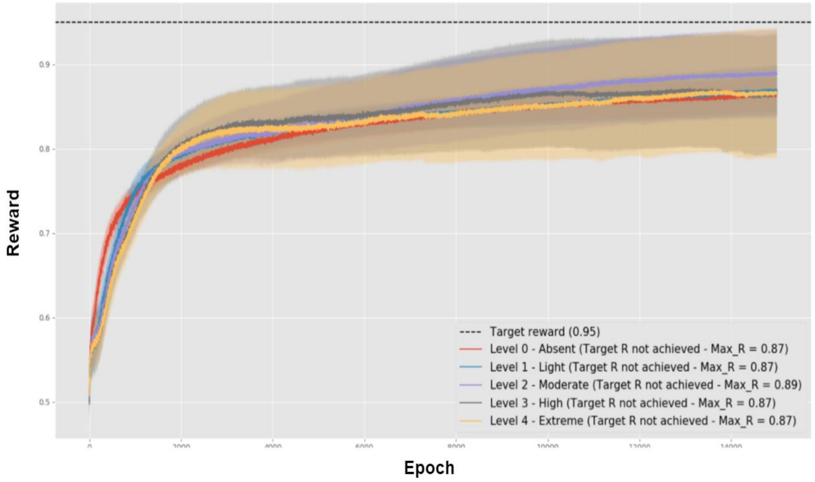

Figure S8. Reward per epoch in the task task involving the size category and high computational resources, of the five models involving different UL/RL levels, averaged over the models using a given level. Shaded areas represent the curves standard deviations.

Table S2 shows the post-hoc tests with the Bonferroni correction. The tests are grouped for each specific combination of the three main conditions, that is, computational resources (2 conditions), sorting rule used in the task (3 conditions), and reward contribution (5 conditions).

Sorting rule: Colour, Computational Resources: Low

	**Absent (L0, N = 40)**	**Low (L1, N = 34)**	**Moderate (L2, N = 21)**	**High (L3, N = 38)**	**Extreme (L4, N = 32)**
**Absent (L0)**	//	$p > 0.05$ (NS)	$p > 0.05$ (NS)	$p > 0.05$ (NS)	$p > 0.05$ (NS)
**Low (L1)**	//	//	$p > 0.05$ (NS)	$p > 0.05$ (NS)	$p > 0.05$ (NS)
**Moderate (L2)**	//	//	//	$p > 0.05$ (NS)	$p > 0.05$ (NS)
**High (L3)**	//	//	//	//	$p > 0.05$ (NS)
**Extreme (L4)**	//	//	//	//	//

Table S2. The table shows the post hoc multiple comparisons (t-test with Bonferroni correction) of models in case of the colour sorting rule and low computational resources. NS = not significant.

Sorting rule: Shape, Computational Resources: Low

	**Absent (L0, N = 32)**	**Low (L1, N = 44)**	**Moderate (L2, N = 29)**	**High (L3, N = 42)**	**Extreme (L4, N = 39)**
**Absent (L0)**	//	$p < 0.001$	$p < 0.001$	$p < 0.001$	$p < 0.001$
**Low (L1)**	//	//	$p < 0.001$	$p < 0.001$	$p < 0.01$
**Moderate (L2)**	//	//	//	$p > 0.05$ (NS)	$p > 0.05$ (NS)
**High (L3)**	//	//	//	//	$p > 0.05$ (NS)
**Extreme (L4)**	//	//	//	//	//

Table S3. The table shows the post hoc multiple comparisons (t-test with Bonferroni correction) of models in case of the shape sorting rule and low computational resources. NS = not significant.

Sorting rule: Size, Computational Resources: Low

	**Absent (L0, N = 38)**	**Low (L1, N = 35)**	**Moderate (L2, N = 39)**	**High (L3, N = 28)**	**Extreme (L4, N = 41)**
**Absent (L0)**	//	$p < 0.001$	$p < 0.001$	$p < 0.001$	$p < 0.001$
**Low (L1)**	//	//	$p > 0.05$ (NS)	$p > 0.05$ (NS)	$p > 0.05$ (NS)
**Moderate (L2)**	//	//	//	$p > 0.05$ (NS)	$p > 0.05$ (NS)
**High (L3)**	//	//	//	//	$p > 0.05$ (NS)
**Extreme (L4)**	//	//	//	//	//

Table S4. The table shows the post hoc multiple comparisons (t-test with Bonferroni correction) of models in case of the size sorting rule and low computational resources. NS = not significant.

Sorting rule: Colour, Computational Resources: High

	**Absent (L0, N = 39)**	**Low (L1, N = 33)**	**Moderate (L2, N = 31)**	**High (L3, N = 20)**	**Extreme (L4, N = 43)**
**Absent (L0)**	//	$p > 0.05$ (NS)	$p < 0.001$	$p > 0.05$ (NS)	$p < 0.01$
**Low (L1)**	//	//	$p < 0.001$	$p > 0.05$ (NS)	$p < 0.05$
**Moderate (L2)**	//	//	//	$p < 0.001$	$p < 0.01$
**High (L3)**	//	//	//	//	$p > 0.05$ (NS)
**Extreme (L4)**	//	//	//	//	//

Table S5. The table shows the post hoc multiple comparisons (t-test with Bonferroni correction) of models in case of the colour sorting rule and high computational resources. NS = not significant.

2.2 Reconstruction error and information stored

In this section we explain why the reconstruction errors of the DBN reported in the main text can be considered a measure of the information on the input patterns

Sorting rule: Shape, Computational Resources: High

	Absent (L0, N = 41)	Low (L1, N = 35)	Moderate (L2, N = 33)	High (L3, N = 29)	Extreme (L4, N = 33)
**Absent (L0)**	//	$p < 0.001$	$p < 0.001$	$p < 0.001$	$p > 0.05$ (NS)
**Low (L1)**	//	//	$p > 0.05$ (NS)	$p > 0.05$ (NS)	$p < 0.001$
**Moderate (L2)**	//	//	//	$p > 0.05$ (NS)	$p < 0.001$
**High (L3)**	//	//	//	//	$p < 0.05$
**Extreme (L4)**	//	//	//	//	//

Table S6. The table shows the post hoc multiple comparisons (t-test with Bonferroni correction) of models in case of the shape sorting rule and high computational resources. NS = not significant.

Sorting rule: Size, Computational Resources: High

	Absent (L0, N = 24)	Low (L1, N = 30)	Moderate (L2, N = 35)	High (L3, N = 29)	Extreme (L4, N = 29)
**Absent (L0)**	//	$p > 0.05$ (NS)	$p > 0.05$ (NS)	$p > 0.05$ (NS)	$p > 0.05$ (NS)
**Low (L1)**	//	//	$p > 0.05$ (NS)	$p > 0.05$ (NS)	$p > 0.05$ (NS)
**Moderate (L2)**	//	//	//	$p > 0.05$ (NS)	$p > 0.05$ (NS)
**High (L3)**	//	//	//	//	$p > 0.05$ (NS)
**Extreme (L4)**	//	//	//	//	//

Table S7. The table shows the post hoc multiple comparisons (t-test with Bonferroni correction) of models in case of the size sorting rule and high computational resources. NS = not significant.

retained by this component of the models. Restricted Boltzmann Machines and Deep Belief Networks are generative models able to store the joint probability between an input and the consequent hidden layer activation [2, 3]. This property makes these models able to execute a dimensional reduction of input patterns [4] and to 'generate' such input patters based on an inverse spread of activation spread from a hidden layer towards the visible layer. Due to the difficulty of meaningfully activating the distributed representations within the hidden layers in a direct way, a typical way to exploit this generativity property also followed here is to precede the hidden-visible activation spreading by a standard visible-hidden activation. This allows the computation of the *reconstruction error*, corresponding to the difference between an input pattern and the corresponding reconstruction. This error is relevant as it represents a measure of the information that the system has retained on the input pattern.

2.3 Internal representations analysis: PCA and K-means details

In the main test we illustrated the results obtained on average over whole classes of simulations. Here we show the outcome of the PCA (principal component analysis) and K-means analyses exemplifying the results within each class. In particular, we considered examples that were more aligned with the average scores of the classes as they should be more representative of the classes themselves.

Tables S8, S9, and S10 show the cumulative explained variance of the the PCA in correspondence to a growing number of principal components. The plots presented in the main text had an $n = 2$ corresponding to the first two principal components. This value is acceptable because it is almost always higher than the median cumulative explained variance and at the same time allowed us to plot the components of the reconstructed images. An interesting feature that emerges from the values is that with a higher value of RL the 'elbow' of the curves represented by the numbers reported in the tables become sharper. This is in line with the fact that with a higher RL contribution the images tend to be increasingly clustered into groups corresponding to the actions to be returned while the task-irrelevant features are discarded, thus needing less components to be represented.

Tables S11, S12, S13 show the silhouette values of the k-means algorithm

PCA cumulative variance explained
(Sorting rule: Colour)

	Absent (L0)	Low (L1)	Moderate (L2)	High (L3)	Extreme (L4)
**N = 1**	0.39	0.48	0.67	0.63	0.64
**N = 2**	**0.62**	**0.76**	**0.99**	**0.99**	**0.99**
**N = 3**	0.74	0.86	1	1	1
**N = 4**	0.81	0.91	1	1	1
**N = 5**	0.85	0.94	1	1	1
**N = 6**	0.89	0.96	1	1	1
**N = 7**	0.91	0.97	1	1	1
**Median**	0.81	0.85	1	1	1

Table S8. Cumulative explained variance (CEV) of the PCA run over the reconstructed images of the models, in the case of the colour sorting rule and low computational resources. The $n = 2$ CEV values are highlighted in bold.

PCA cumulative variance explained
(Sorting rule: Shape)

	Absent (L0)	Low (L1)	Moderate (L2)	High (L3)	Extreme (L4)
**N = 1**	0.39	0.48	0.53	0.73	0.67
**N = 2**	**0.64**	**0.74**	**0.87**	**0.94**	**0.98**
**N = 3**	0.76	0.83	0.99	0.99	1
**N = 4**	0.82	0.89	0.99	1	1
**N = 5**	0.85	0.93	1	1	1
**N = 6**	0.88	0.94	1	1	1
**N = 7**	0.91	0.96	1	1	1
**Median**	0.82	0.89	1	1	1

Table S9. Cumulative explained variance (CEV) of the PCA run over the reconstructed images of the models, in the case of the shape sorting rule and low computational resources. The $n = 2$ CEV values are highlighted in bold.

PCA cumulative variance explained
(Sorting rule: Size)

	Absent (L0)	Low (L1)	Moderate (L2)	High (L3)	Extreme (L4)
**N = 1**	0.39	0.47	0.61	0.70	0.60
**N = 2**	**0.63**	**0.76**	**0.91**	**0.90**	**1**
**N = 3**	0.75	0.86	0.99	0.99	1
**N = 4**	0.81	0.92	1	1	1
**N = 5**	0.85	0.95	1	1	1
**N = 6**	0.88	0.96	1	1	1
**N = 7**	0.90	0.97	1	1	1
**Median**	0.81	0.92	1	1	1

Table S10. Cumulative explained variance (CEV) of the PCA run over the reconstructed images of the models, in the case of the size sorting rule and low computational resources. The $n = 2$ CEV values are highlighted in bold.

corresponding to different K values establishing the number of the searched classes. The tables show that the the highest silhouette values tend to correspond to $K = 4$, the value

used in the analyses reported in the main text. This value is relevant as it corresponds to the number of attributes in each category and to which the model has to assign a different action (colour: red, green, blue, yellow; form: square, circle, triangle, bar; size: large, medium-large, medium-small, small). It is also interesting to observe that the best silhouette value is more highly differentiated from other values in correspondence to higher levels of RL contribution: this agrees with the fact that in these conditions the model tends to encode features that are more closely related to the actions.

K-means Silhouette values
(Sorting rule: Colour)

	**Absent (L0)**	**Low (L1)**	**Moderate (L2)**	**High (L3)**	**Extreme (L4)**
**K = 2**	0.47	0.50	0.71	0.55	0.63
**K = 3**	0.56	0.61	0.91	0.80	0.80
**K = 4**	**0.64**	**0.65**	**1**	**1**	**1**
**K = 5**	0.66	0.63	0.86	0.69	0.91
**K = 6**	0.69	0.64	0.53	0.53	0.78
**K = 7**	0.73	0.67	0.52	0.27	0.66
**K = 8**	0.72	0.66	0.44	0.42	0.60
**Mean**	0.64	0.62	0.71	0.61	0.77

Table S11. The table shows the K-means silhouette values of models in case of colour sorting rule and low computational resources. The K = 4 silhouette values are highlighted in bold

K-means Silhouette values
(Sorting rule: Shape)

	**Absent (L0)**	**Low (L1)**	**Moderate (L2)**	**High (L3)**	**Extreme (L4)**
**K = 2**	0.44	0.51	0.55	0.69	0.63
**K = 3**	0.53	0.61	0.74	0.84	0.82
**K = 4**	**0.63**	**0.68**	**0.94**	**0.99**	**0.92**
**K = 5**	0.65	0.66	0.99	0.98	0.98
**K = 6**	0.67	0.66	0.98	0.96	0.98
**K = 7**	0.70	0.66	0.80	0.93	0.71
**K = 8**	0.66	0.63	0.80	0.94	0.65
**Mean**	0.61	0.63	0.83	0.90	0.81

Table S12. The table shows the K-means silhouette values of models in case of shape sorting rule and low computational resources. The K = 4 silhouette values are highlighted in bold

References

1. Hinton GE. Training products of experts by minimizing contrastive divergence. Neural computation. 2002;14(8):1771–1800.

2. Hinton GE, Osindero S, Teh YW. A fast learning algorithm for deep belief nets. Neural computation. 2006;18(7):1527–1554.

3. Hinton GE. A practical guide to training restricted Boltzmann machines. In: Neural networks: Tricks of the trade. Springer; 2012. p. 599–619.

K-means Silhouette values
(Sorting rule: Size)

	Absent (L0)	Low (L1)	Moderate (L2)	High (L3)	Extreme (L4)
**K = 2**	0.47	0.53	0.65	0.70	0.75
**K = 3**	0.55	0.63	0.87	0.83	0.86
K = 4	**0.64**	**0.72**	**0.95**	**1.0**	**0.99**
**K = 5**	0.64	0.71	0.97	0.87	0.98
**K = 6**	0.66	0.71	0.94	0.71	0.69
**K = 7**	0.68	0.72	0.97	0.48	0.66
**K = 8**	0.68	0.72	0.95	0.48	0.49
**Mean**	0.62	0.68	0.90	0.73	0.78

Table S13. The table shows the K-means silhouette values of models in case of size sorting rule and low computational resources. The K = 4 silhouette values are highlighted in bold

4. Hinton GE, Salakhutdinov RR. Reducing the dimensionality of data with neural networks. science. 2006;313(5786):504–507.
