# Supplementary material for: Integrating unsupervised and reinforcement learning in human categorical perception: A computational model
Source: PLoS One. 2022 May 10;17(5):e0267838. doi: 10.1371/journal.pone.0267838 (PMC9089926; doi:10.1371/journal.pone.0267838)
Supplement: Attachment — Submitted filename: Response to Reviewers.pdf [file pone.0267838.s002.pdf]

Revision

Giovanni Granato, Emilio Cartoni, Federico Da Rold, Andrea Mattera, Gianluca Baldassarre;
4 March 2022

Revision introduction

The document is organised as follows:

- The observations of the Reviewers are reported in **bold**.
- The replies are reported in “plain text”.

Editor’s comments and authors’ replies

OBSERVATION 1

Dear Dr. Granato,

Thank you for submitting your manuscript to PLOS ONE. After careful consideration, we feel that it has merit but does not fully meet PLOS ONE’s publication criteria as it currently stands. Therefore, we invite you to submit a revised version of the manuscript that addresses the points raised during the review process.

Please submit your revised manuscript by Mar 19 2022 11:59PM. If you will need more time than this to complete your revisions, please reply to this message or contact the journal office at plosone@plos.org. When you're ready to submit your revision, log on to https://www.editorialmanager.com/pone/ and select the 'Submissions Needing Revision' folder to locate your manuscript file.

Please include the following items when submitting your revised manuscript: A rebuttal letter that responds to each point raised by the academic editor and reviewer(s). You should upload this letter as a separate file labeled 'Response to Reviewers'.
A marked-up copy of your manuscript that highlights changes made to the original version. You should upload this as a separate file labeled 'Revised Manuscript with Track Changes'.
An unmarked version of your revised paper without tracked changes. You should upload this as a separate file labeled 'Manuscript'.
If you would like to make changes to your financial disclosure, please include your updated statement in your cover letter. Guidelines for resubmitting your figure files are available below the reviewer comments at the end of this letter.

We look forward to receiving your revised manuscript.

Kind regards,

Frederic Alexandre

Academic Editor
PLOS ONE

REPLY 1

Dear Prof. Frederic Alexandre,

Thank you for giving us the opportunity to resubmit this manuscript. We were glad to see that you and the reviewers saw potential in our manuscript. We have now revised the manuscript in accordance with the reviewers' suggestions.

We feel that the manuscript has improved in impact and clarity as a result of such changes. We hope that the manuscript has become more adequate for publication. We provide below a full list of the changes we have made.

Sincerely,
Giovanni Granato
Emilio Cartoni
Federico Da Rold
Andrea Mattera
Gianluca Baldassarre

Additional Editor’s comment

OBSERVATION 2

Both reviewers have commented on the paper and advise a major revision. More specifically, claims in the abstract and introduction should better correspond to what is reported in the Limitations part. In addition and as requested by the reviewers, some details about the model should be added for clarity. Thank you for considering these points.

REPLY 2

Thank you for this summarising comment. We revised the manuscript to clarify the focus of this work in many sections. Moreover we added many details (both additional figures and textual descriptions) regarding the methodology we adopted to build the computational model.

Reviewer 1 comments and authors' replies

OBSERVATION 0

In the present article, the authors propose a model for human categorical perception and the underlying learning process. They train a multi-layer artificial neural network to perform an image-sorting task. In this framework, they show that a combination of Hebbian unsupervised learning and reward-driven learning in a given layer of the network is significantly more efficient (better final performance) than Hebbian learning alone or reward-driven learning alone.

While the main result of the paper may be interesting, the incomplete description of the model's implementation makes it difficult to understand the breadth of the results or to reproduce it. The conditions in which the model is tested are not very well described and it is difficult to know if they can be generalized to other tasks and/or representations of the categorical learning system representation. Finally, the model of the brain networks involved in categorical perception is quite sophisticated (many layers and several learning algorythms in different layers) while the representation of elementary computational units is simplistic and based on previous frameworks developed in machine learning. It is thus unclear how the articulation between various components of the artificial network may be linked with natural brain processes underlying categorical perception, as suggested by the title and abstract.

Overall, I would be more confident in my opinion on this work if the model's implementation, the task and the results were described in full details. Moreover, the results of the model could be discussed in relation to brain processes in the discussion rather than being interpreted in terms of categorical learning in brain networks in the abstract and title.

REPLY 0

Thanks for this overall feedback. We have made several changes to the manuscript to address the issues raised. In particular, we have added many methodological details regarding the task protocol, the model implementation and the experimental conditions from which we obtained the final results.
Moreover, we added a clear description of our approach to the model description and building. This approach, commonly adopted by us and many computational communities, follows specific sequential steps: (1) description of the brain systems supporting the investigated processes and novel theoretical proposal, (2) introduction to the neuro-inspired rationale and implementation details of the model, (3) testing of the model with a neuropsychological-like task, (4) discussion of the relationships between our results and neurocognitive human processes in case of healthy and clinical conditions.

- Major observations

OBSERVATION 1

Abstract:

It's not clear if all assertions from the abstract are coming from the authors results or if they come from previous studies. In particular, the sentence "Indeed, an excessive unsupervised learning contribution tends to underrepresent task-relevant features while an excessive reinforcement learning contribution tends to initially learn slowly and then to incur in local minima." There is no result about local minima and this is only evoked in the discussion as a possible interpretation of the results. If so, it should not stand as an assertion in the abstract.

REPLY 1

We have removed the specific assertions in the abstract regarding local minima, further adapting the text to better fit our concrete results.

OBSERVATION 2

Figure 1 : Instead of simply showing examples of visual elements, a graphical description of the task would help the reader understand what is modelled here.

REPLY 2

We added a further sub-figure that represents the task protocol. In particular the figure shows (1) the distinction between pre-task processes (set of task conditions) and task performance (trials sequence), (2) the input given to the model in each trial, (3) the intra-trial sequence of the task phases (i.e. from the input processing to the perceptual/motor adaptation).

OBSERVATION 3

Methods:
1.1: The task description is not clear. While the authors explain how the task is linked to other tasks, it is not clear what exact task is modelled here and how it is represented/run in the model.

REPLY 3

We now clearly described each detail of the task protocol. In particular we described the protocol sections (pre-task and task performance) and each intra-trial phase. We also clearly

provide many details of the interaction between the model and the experimental protocol such as dataset, inputs/output of trials, score computation.

OBSERVATION 4

1.2: This part does not include a description of the model is not full and it is unclear why it belongs to the methods as is not linked to the model's implementation.

The extensive comparison between the actual model and its interpretation rather belongs to the discussion of the paper. As an example, the authors explain first how the model may be "coherent with the theoretical framework of embodied perception", even before explaining what the model consists in. Figure 2, summarizing the parallel between the model and real-world brain structures (again rather a discussion point) stands before any description of the model. Same in the text (e.g. "The architecture and learning processes of the model are inspired by the interactions between brain cortical and sub-cortical macro-systems"). As a result, the reader is left with speculations rather than being able to understand what the authors did.

Discussing future work also belongs to the discussion rather than methods ("Future works will make the learning mechanisms of the model more gradual along the sensory-motor hierarchy").

In this part, the authors simply refer to Figure 3 (schematics of the model's component) and provide a qualitative description of each component, again making the hypothetical link to biology and other work rather than describing the implementation they performed (e.g. "This component is based on a neural network that receives visual inputs and performs information abstraction, mimicking the brain visual system.").

It is not clear why Figure 3 is different from Fig 4 and why part 1.2 precedes the real methods part (1.3 describing model implementation). Why should the similarity to other works and biological network not rather be in the discussion? Because it stands before the implementation of the model, it adds rather confuses the reader in my opinion.

REPLY 4

We understood and agree on the potential confusion of the reader about our approach in the manuscript organisation. To address this issue we now have described our theory-driven approach at the start of the methods section, indicating that we first describe the neuro-inspired rational of the model and then the model algorithmic details. Furthemore, we clarify the role of section 1.3 adopting a new title "Neuro-inspired underpinnings of the model: main learning processes and key components".
This approach is considered a standard in many computational communities and we successfully adopted it in our past publications (e.g. Granato and Baldassarre, 2021; Granato et al., 2020).

We would adopt this approach in our manuscripts because we think that it makes the publication more readable for many scientists from experimental/clinical fields (e.g. cognitive neuroscientists, clinical neuropsychologists, psychiatrists), computational fields (e.g. computational neuroscientists/neuropsychologists) and technological fields (e.g. machine learners/roboticists). In particular each scientist can focus on a specific “analysis layer” of our model, from a more abstract (neuro-inspired underpinning) to a more algorithmic (training equations in the manuscript and parameters details in supplementary materials).

If the reviewer does not agree with our solution we could adopt an alternative one, for example inserting a section between introduction and methods that explains the neuro-inspired rationale of the model.

OBSERVATION 5

1.3 The model implementation is given in this part. Still, some details are missing to fully understand the mode without going to the cited publication:

- How is the RBM ‘trained’? What is the ‘reconstructed visible activation’? Does an RBM have 2 layers or more? What is data and model (reconstructed) then? In other words, if one RBM is 2 layers (v and h), how can it have a data layer and a model layer? I did not understand. A figure representation of the component could help understanding its structure and the various names used.

- Same problem with the second RBM.

- Motivational component. The predictor module is not fully described here.
Moreover, the description is hard to follow due to the lack of figure.

- Motor component: is it an RBM as well? Or a single layer? A representation would help

- A global view of the network with all layers and names would help the reader follow the model’s description and implementation.

REPLY 5

We now better clarify in the text the structure of these components and the training of the RBM. Moreover, we updated the figure 5 (computational schema of the model), substituting the labels of the predictor and the motor components respectively with “MLP” (Multi-layer perceptron) and “SLP” (Single-layer perceptron). We also added a label on the side of the MLP that identifies the hidden layer of the network. These acronyms are commonly adopted in the modelling and ML communities (e.g. MLP for “Multi-layer Perceptron”) and we have made them clear in the figure caption.

In addition to these adjustments, we added to supplemental materials two further figures. Figure S1 shows a raw global view of the model components implemented with neural networks, reporting the number and the type of units of each layer. Figure S2 proposes a graphical description of the common method used to train a Restricted Boltzmann Machine with Contrastive Divergence. We would prefer to add these figures to supplemental materials to keep the manuscript more light and easily readable.

OBSERVATION 6

Results:

Just as the methods section, the results section lacks precision. As an example, it starts with “We tested the model with different conditions of the sorting task, each involving one out of three sorting rules. For example, …”. The different conditions are never described unambiguously (unless I missed it).

Another example in the following sentences: “Note that 50 units were sufficient to allow the system to fully encode the image features, as shown by a preliminary test indicating a close-to-null reconstruction error.” This result is not shown anywhere (neither the result of the reconstruction, nor the error value, nor how the error is computed).

The clear and full description of the task begin still missing, it is difficult to understand the beginning of the results description.

The results talk about performance in groups not being defined before.

REPLY 6

We have adjusted the initial part of the results to address this issue. We hope that the rationale of our tests now appears clearer, as well as the task conditions and the groups tested.

OBSERVATION 7

Discussion:

The 10 pages discussion appears a bit excessive given the relatively thin results section and the application of the learning algorithm in a very tight context.

REPLY 7

We agree with this consideration and we have made an effort to summarise the whole section, removing many redundant explanations and passing from 10 pages to 9 pages. Also consider that the Reviewer 2 has requested the addition of a discussion on some aspects

linking our work and the ML field, hence requiring the addition of further text that is included in the 9 pages, so the cut done to the previous text is heavier than it might appear.

On this issue, we would like to highlight that despite our results being limited, our theoretical treatments are quite wide in scope and the model results have thus allowed us to support many comparisons with different scientific fields. For example, the section of the main contributions (now labelled “Main contributions, clinical relevance and technological implications”) proposes many links with experimental cognitive neuroscience/neuropsychology/psychiatry on the one hand and machine learning/robotics on another. Furthemore, the discussion is so extended also because it includes a review of the computational models of human categorical perception summarising the literature and highlighting that our model is comparable with the state-of-the-art. Finally, the section “Limits and future directions” is particularly extended because we are aware of the model limitations but also of the many promising future directions that can be developed on the basis of this first “proof-of-concept”.

Reviewer 2 comments and authors’ replies

OBSERVATION 0

The authors present a systems-level categorical perception model and present computational simulation results establishing that a balanced mix of unsupervised and reinforcement learning leads to emergence of categorical perception. The authors present a simple system level approach that introduces a balancing of unsupervised learning with reinforcement learning, and as the authors claim, this could be a proof-of-concept architecture that could give clinical insights in autism. The paper is also well written and explains the architecture of the model and its limitations well.

REPLY 0

Thanks for the positive comments.

# - Major observations

OBSERVATION 1

1. The authors put forward the argument that dopamine/RL is involved in categorical perception? Is there any empirical evidence to back up this claim?

REPLY 1

We clarified in the text that our theoretical proposal is based on many studies regarding the relationship between the dopamine-related DA and categorisation processes, of which categorical perception is an instance. Our theoretical proposal (categorical perception and DA-related RL signals) is based on the following inferential reasoning: “experimental evidence suggests that DA-related RL signals influence categorisation processes and sensory representations, hence they could influence the emergence of categorical perception”. Therefore, we adjusted the text to be more coherent with experimental evidence. Finally, we insert a reference to a review (Lim et al, 2014) that includes an extended analysis regarding the role of the dopamine-related RL in sensory cortices and categorisation processes.

OBSERVATION 2

2. Though the task is adaptive categorical perception, the paper reduces it to a clustering study and shows representations that are optimal for each task in isolation.

There is no adaptation in the algorithm itself and it does not solve these tasks simultaneously. The time scales of the reinforcement learning where it is learnt though a policy gradient for each task does not permit rapid adaption and the authors point out this in the limitations section that it would cause catastrophic forgetting. The reader does not know until the limitations section that the proposed model/simulations do not address the adaptive categorical perception. Hence the abstract, motivation, introduction could be misleading to the reader who might be eagerly waiting for an algorithmic solution to the adaptive categorical perception. This needs to be spelt out before the limitations. This can be done in two ways: i) the computational model details as well as preliminary solution for the adaptive version could be included or ii) the manuscript could focus on the categorical perception solution with balanced UL+RL and present the adaptive version as a possible extension as a separate section in the discussion. The authors could decide. Not presenting the model/simulations for adaptive version does not mitigate the importance of the current model and results.

REPLY 2

We have solved the semantic conflict, underlining in the text that we focus on categorical perception where the sorting rule does not change. In particular, we removed the terms “adaptive” that created the conflict in Abstract and Introduction. Moreover, we expanded the two paragraphs “transfer learning skills and generalisation analysis” and “Multi rules categorisation and catastrophic forgetting” in Discussion, proposing a possible solution that leads to (1) testing this model also in case of adaptive categorical perception and (2) building a new model that supports adaptive categorical perception. We preferred adopting this solution, suggested by the reviewer, because we are convinced that this type of adaptation represents an important scientific topic we want to investigate in the future starting from this proof-of-concept.

OBSERVATION 3

3. In section 1.2, it is mentioned that the architecture is coherent with the theoretical framework of embodied perception. However, I feel the motor component is not central to the model and acts as an addendum, it is not clear how this model develops a coherent link with embodied cognition other than its simplistic motor component.

REPLY 3

We better clarify in the Methods section the motives for which we propose that the learning processes are coherent with the theories of embodied perception. In particular, while admitting that we do not have realistic motor components/actuators and an full embodied model, we highlighted that in the simulation the virtual world affects the perceptual states of the agent, in turn affecting its response toward the world in a circular loop. For this reason, the categorical perception emerges in the model in a feedback-dependent manner through the clues provided by the world. According to some views with which we agree (Da Rold, 2018) this represents a key feature of embodied perception. Note that in the Discussion section there is a specific paragraph highlighting this limited link with embodiment and a possible solution (robotic implementation) to move towards a more realistic embodiment.

OBSERVATION 4

4. The paper puts forward RBM as the unsupervised component, the authors could have a discussion on other possible architectures that could do adaptive categorical perception, for example a VAE or a conditional VAE. They could discuss the relative merits and demerits.

Re: Karol Gregor, George Papamakarios, Frederic Besse, Lars Buesing, Theophane Weber: Temporal Difference Variational Auto-Encoder. ICLR 2019

REPLY 4

We insert in Discussion (section “Main contributions, clinical relevance and technological implications”) a computational deepening that focuses on the comparison between our approach (a DBN trained with a novel learning rule) and common ML approach in representation learning (e.g. VAE). Although we think the proposed model is still a proof-of-concept, we think that our architecture and learning rules also represent a starting point for future ML and robotics architectures.

OBSERVATION 5

5. The paper claims that this intermixing of UL & RL in the work could lead to more adaptive architecture and is relevant to machine learning. Recent works on this could be explained in a section but it also has to be pointed out RL has a long history with representation learning and interaction of UL & RL.

REPLY 5

We insert in Discussion (section “Main contributions, clinical relevance and technological implications”) a premise and specific references regarding the tradition of RL and representation learning, including the UL/RL interaction.

OBSERVATION 6

6. The paper puts forward the argument that the interaction of UL & RL is necessary for adaptive categorical perception and other sensory processing and rules out the possibility that adaptive representations will not arise through RL alone. Of course some of these labels are semantics but there have been architectures driven purely by reward related learning that have shown complex adaptive processing. For example. the meta RL as a prefrontal cortex paper (Ref below) arrives at adaptive dynamics purely by reward related feedback. It might be misleading to point out interaction between UL+RL is the only way for adaptive perception to take place and it could do with the simplified architecture of the model.

Ref: Wang, J.X., Kurth-Nelson, Z., Kumaran, D. et al. Prefrontal cortex as a meta-reinforcement learning system. Nat Neurosci 21, 860–868 (2018). https://doi.org/10.1038/s41593-018-0147-8

Additional references that the authors could consider:

David Ha, Jürgen Schmidhuber 2018). World Models. CoRR abs/1803.10122.

Doya, K. (1999). What are the computations of the cerebellum, the basal ganglia and the cerebral cortex? Neural Networks, 12(7-8), 961–974. https://doi.org/10.1016/S0893-6080(99)00046-5

REPLY 6

We now more clearly stated that our solution (UL/RL integration), despite being based on scientific evidence from different research fields, is only a possible explanation of brain processes supporting perceptual representation learning. In particular, we insert this premise and the references suggested by the reviewer in the text explaining the neuro-inspired rationale at the basis of the model development. Interestingly, our recent theoretical proposal on “Superlearning” cited in our paper starts from the paper of Doya (1999). Moreover, Ha and Schmidhuber (2018) state that the pure UL of VAE is affected by a potential “inefficient” representation learning not favouring the emergence of task-directed representations. This last statement guided our model conception, design and building.

# - Minor observations

OBSERVATION 7

Authors refer to a 2D input stimulus as "parallelopiped", perhaps "rectangle" is more appropriate?

REPLY 7

We substituted the terms as suggested by the reviewer. Indeed the previous term referred to a 3D object but our protocol provides 2D images.
